# Low-level auricular vagus nerve stimulation lowers blood pressure and heart rate in paroxysmal atrial fibrillation patients: a self-controlled study

**DOI:** 10.3389/fnins.2025.1525027

**Published:** 2025-01-29

**Authors:** Yu Jiang, Di Su, Jingwen Xiao, Wenbo Cheng, Yuemei Hou, Yan Zhang

**Affiliations:** ^1^Department of Cardiovascular Medicine, Fuzhou First Hospital Affiliated With Fujian Medical University, Fuzhou, China; ^2^Cardiovascular Disease Research Institute of Fuzhou City, Fuzhou, China; ^3^Teaching Experimental Center for Pharmacy, School of Pharmaceutical Sciences, Shanghai Jiao Tong University, Shanghai, China; ^4^Baoshan Branch, Ren Ji Hospital, School of Medicine, Shanghai Jiao Tong University, Shanghai, China; ^5^Department of Geriatrics, Shanghai University of Medicine & Health Sciences Affiliated Sixth People's Hospital South Campus, Shanghai, China

**Keywords:** paroxysmal atrial fibrillation, low-level auricular vagus nerve stimulation, autonomic nervous system, blood pressure and heart rate, feasibility and safety

## Abstract

**Objective:**

To investigate the effects of low-level auricular vagus nerve stimulation (LL-aVNS) on blood pressure and heart rate in patients with paroxysmal atrial fibrillation.

**Methods:**

A total of 22 patients with paroxysmal atrial fibrillation diagnosed in Fuzhou First General Hospital Affiliated with Fujian Medical University from September 2021 to December 2022 were selected and given LL-aVNS treatment based on the original unchanged drug treatment for 4 weeks. The systolic blood pressure (SBP), diastolic blood pressure (DBP), heart rate maximum (HRmax), heart rate minimum (HRmin), left ventricular ejection fraction (LVEF), left atrial diameter (LAD), Atrial Fibrillation Severity Scale (AFSS) symptom subscale, and Memorial Symptom Assessment Scale-Heart Failure (MSAS-HF) before and after the treatment were observed and compared. In addition, adverse effects of the LL-aVNS procedure and 6-month follow-up were recorded.

**Results:**

SBP, DBP, and HRmin were lower after the treatment than before the treatment (*p* < 0.05); AFSS symptom subscale scores and MSAS-HF scores after the treatment were lower before the treatment (*p* < 0.05); itching of the skin was observed in one case during the course of LL-aVNS; and two patients were hospitalized for acute exacerbation of chronic heart failure between 4 months and 6 months after the treatment.

**Conclusion:**

LL-aVNS in patients with paroxysmal atrial fibrillation can assist in controlling blood pressure and heart rate, effectively relieving symptoms, and the treatment process is safe and feasible.

## 1 Introduction

Atrial fibrillation (AF) is one of the most common clinical arrhythmias associated with disorganized atrial electrical activity and ineffective atrial contraction, leading to high rates of disability and mortality and posing a serious threat to the quality of life and health of patients (Hindricks et al., 2021; Shi et al., 2022; Kornej et al., 2020). Paroxysmal AF is defined as AF that is spontaneous or terminated by treatment within 7 days of onset (Hindricks et al., 2021). According to the Framingham Heart Study (FHS), the prevalence of AF has tripled globally over the past 50 years (Schnabel et al., 2015). A Realistic Global Survey of AF Patients (RealiseAF) showed that the prevalence of paroxysmal AF was 26.5%, with a further progression to persistent and permanent AF (Chiang et al., 2012).

Nowadays, managing paroxysmal atrial fibrillation relies heavily on pharmacologic and surgical approaches, of which the latest guidelines upgrade the level of recommendation after catheter ablation (Liu et al., 2023; Joglar et al., 2024). For patients who do not wish to undergo catheter ablation, scientists are exploring innovative clinical treatment strategies. The cardiac autonomic nervous system is deemed a key controller in the emergence and progression of AF, playing a pivotal role in initiating paroxysmal AF (Shen et al., 2011). Consequently, in recent years, the emphasis has shifted to low-level auricular vagus nerve stimulation (LL-aVNS; Sohinki and Stavrakis, 2020; Stavrakis et al., 2015; Stavrakis et al., 2017). LL-aVNS serves as a non-intrusive therapeutic method for electrically stimulating the area where the ear vagus nerve is distributed, using electrical impulses of a specific intensity (Murray et al., 2016). Animal studies have found that vagal nerve stimulation can inhibit AF, reverse atrial remodeling, and shorten the effective atrial occlusion period in animal models of rapid atrial pacing (Lu et al., 2016). Relevant clinical studies have demonstrated that LL-aVNS inhibits the elevated level of inflammation and reduces the load of atrial fibrillation in patients with paroxysmal AF (Stavrakis et al., 2020; Kulkarni et al., 2021).

Research has demonstrated that elevated blood pressure heightens the risk of atrial fibrillation (AF), even when readings fall within the normal range (Aune et al., 2023). Additionally, findings from another clinical study indicated that a resting heart rate of 80 beats per minute or higher is linked to an increased mortality risk in patients with AF (Han et al., 2024). Irregular heart rhythms and tachycardia may precipitate cardiomyopathy, potentially serving as a mechanism that contributes to heart failure in individuals with AF (Bidaoui et al., 2024). It is noteworthy that autonomic reflexes play a significant role in establishing a hyperadrenergic state within the context of heart failure (Toschi-Dias et al., 2017). Therefore, it is crucial to effectively manage both blood pressure and heart rate in patients with AF.

Currently, the LL-aVNS stimulation parameters (e.g., stimulation frequency, pulse width, current intensity, etc.) used in various clinical studies vary, and in the real world, the treatment of AF cannot be completely relied on LL-aVNS alone. Therefore, this study will focus on the modulation of blood pressure, heart rate, and AF symptoms by LL-aVNS and the follow-up of patients after the treatment, to further explore the effective stimulation regimen of LL-aVNS, and thus confirm the feasibility and safety of LL-aVNS in combination with drugs for the treatment of AF.

## 2 Methods

### 2.1 Study design and ethics

**Figure 1:**
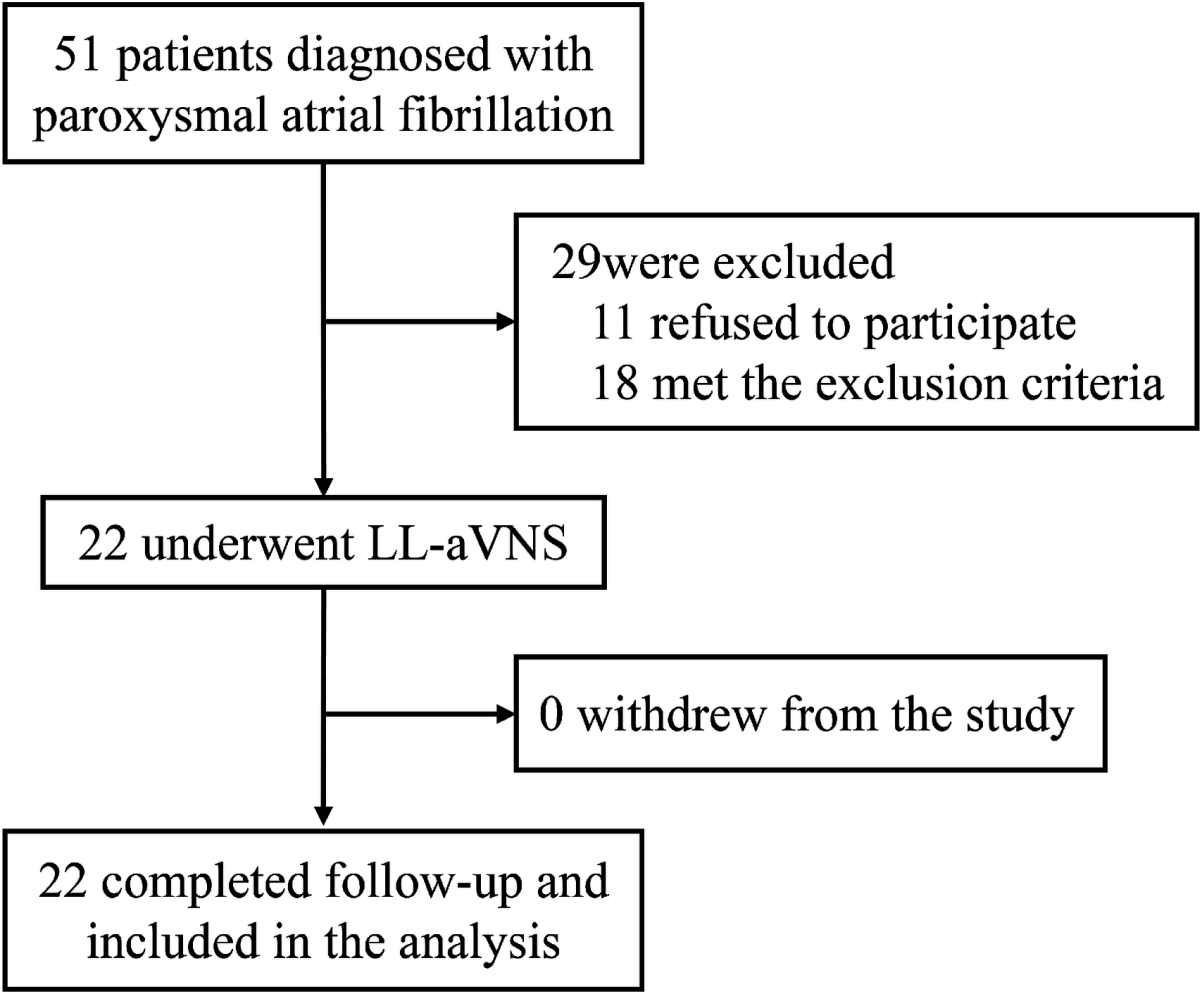
Flowchart of participant involvement in research and follow-up.

A total of 22 patients with paroxysmal AF diagnosed in Fuzhou First General Hospital Affiliated with Fujian Medical University from September 2021 to December 2022 were selected, as shown in Figure 1. Among them, 14 cases were male and 8 cases were female.

The inclusion criteria were: (a) Individuals who meet the diagnostic quasi of paroxysmal AF (based on the 2020ECS/EACTS guidelines for the diagnosis and management of atrial fibrillation); (b) Individuals aged ≥18 years; and (c) The participants provided written informed consent before enrolling in the study and all research procedures were carried out in accordance with the Declaration of Helsinki.

The exclusion criteria were: (a) individuals with atrial thrombus detected by cardiac ultrasound; (b) individuals with pacemaker equipped; (c) individuals with a previous history of stroke; (d) individuals with a previous history of severe wasting disease such as malignant tumor; (e) individuals with psychiatric and psychological disorders; (f) individuals with severe allergic diseases; (g) individuals with other severe physical diseases; and (h) individuals with recent history of trauma.

This study was approved by the Ethical Review of Medical Innovation Research Special Program of Shanghai Science and Technology Innovation Action Plan 2020.

### 2.2 LL-aVNS

**Figure 2:**
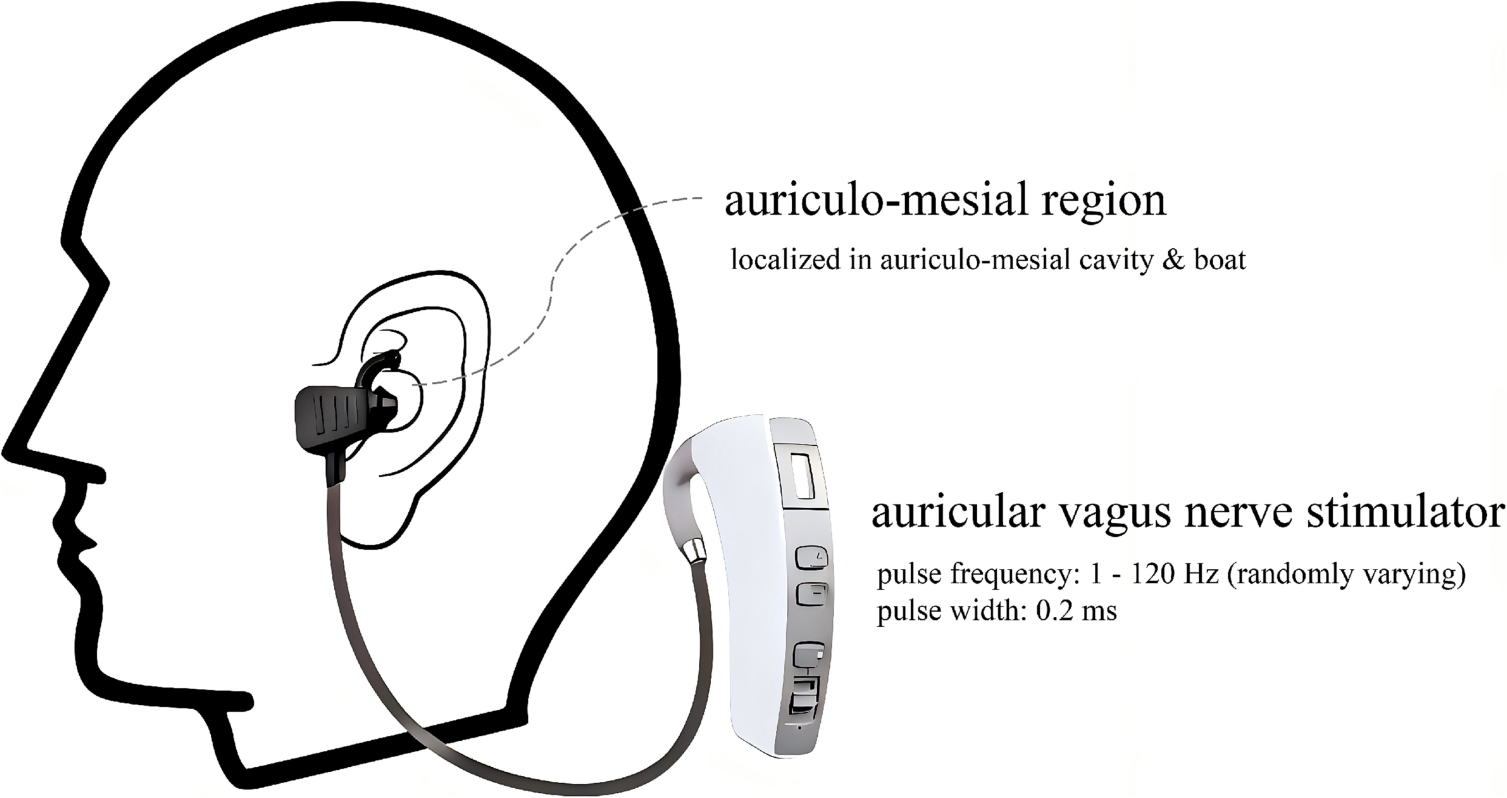
Schematic of the required region and equipment for the LL-aVNS.

The patients with paroxysmal AF included in the study were treated with LL-aVNS for 4 weeks based on the original drug therapy unchanged (Such as Beta blockers, Calcium channel blockers, Statins, and Diuretics). As shown in Figure 2, the LL-aVNS was stimulated using an auricular vagus nerve stimulator (Huatuo brand, TENS-200A, Suzhou Medical Supplies Co., Ltd.) in the auriculo-mesial region (anatomically localized to the auriculo-mesial cavity and the auriculo-mesial boat; Peijing et al., 2013; Haixia et al., 2022). The pulse frequency was chosen to be 1–120 Hz, with random variation, and the pulse width was 0.2 ms. The current intensity threshold was the intensity that could be tolerated without producing pain. The current intensity of each stimulation was 5 mA, 8 mA, 10 mA, 15 mA, and threshold intensity randomized sequential stimulation, the time of different stimulation intensity was 10 min respectively, and the total stimulation time was 50 min, once a day at a fixed time starting at 16:00 PM every day for 4 weeks. Criteria for termination of treatment: (a) individuals who could not tolerate the stimulation; (b) individuals who stopped LL-aVNS on their own; and (c) individuals who showed the presence of the left atrium on ultrasonography or detected thrombus during treatment.

### 2.3 Collection of general information

Before the start of treatment, general information about the patients was collected, including age, height, weight, smoking history, CHA2DS2-VASc score, history of previous diseases, and medications taken.

### 2.4 Measurement of blood pressure and heart rate

BP and HR data were collected for a single treatment session: During the first 1 week of treatment, patients’ blood pressure and heart rate were collected throughout the treatment using Patient Monitor (PHILIPS, GS20, Philips Goldcorp Shenzhen Industrial Co., Ltd.), and systolic blood pressure (SBP), diastolic blood pressure (DBP), maximum heart rate (HRmax), and minimum heart rate (HRmin) were recorded for the 10 min before a single treatment, at different current intensities, and for the 10 min after a single treatment.

BP and HR data were collected before and after 4 weeks of treatment: SBP, DBP, HRmax, and HRmin data were collected from patients for a 24H period on a half-hourly basis using an ambulatory blood pressure recorder (VasoMedical, CB-2304-A, Wuxi Zhongjian Science Instrument Co., Ltd., Wuxi, Jiangsu Province, Jiangsu Province, China) on the day before the start of the 4-week treatment and the day after the end of the 4-week treatment.

### 2.5 Echocardiographic evaluation

Echocardiography was refined before and after 4 weeks of treatment, and left ventricular ejection fraction (LVEF) and left atrial diameter (LAD) were recorded.

### 2.6 Assessment of symptoms

Two scales were used in this study to assess symptoms before and after 4 weeks of treatment. The assessment and collection of the scales were performed by specially trained research nurses. The Atrial Fibrillation Severity Scale (AFSS) symptom scale included 7 common AF symptoms (palpitations, shortness of breath at rest, shortness of breath with activity, exercise intolerance, dizziness, fatigue, and chest pain), with each symptom being expressed on a 6-point Likert scale ranging from none (1 point) to severe (6 points). The Memorial Symptom Assessment Scale for Heart Failure (MSAS-HF) includes 32 physical and psychological symptoms, each of which is scored in terms of symptom occurrence and frequency, severity, and degree of distress (expressed on a Likert scale), ranging from none (1 point) to severe (4 points). Both the AFSS and the MSAS-HF have been validated for use in symptom analysis in clinical studies (Streur et al., 2018; Zhou et al., 2022).

### 2.7 Follow-up visits

**Figure 3:**
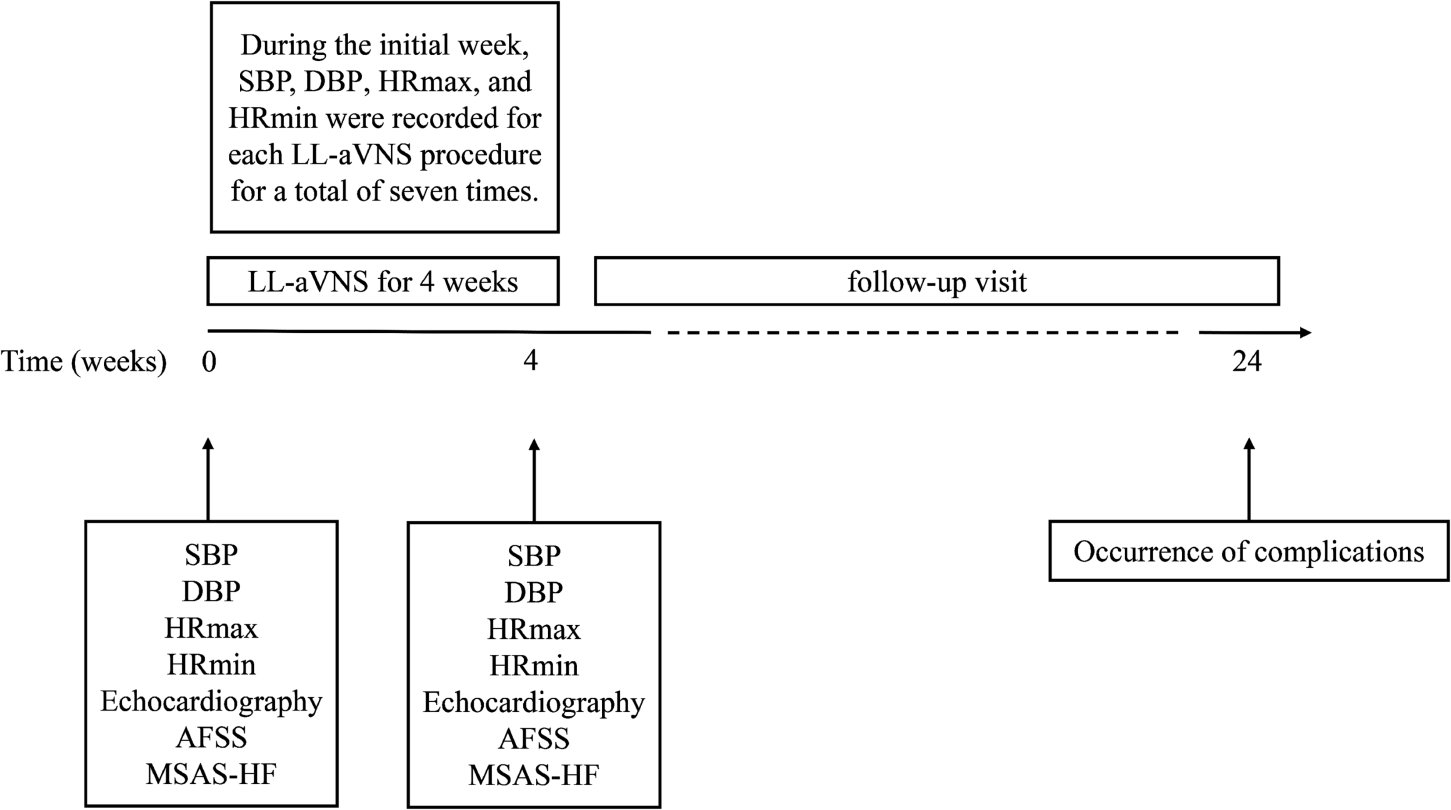
Schematic of the research design timeline.

Follow-up was planned for 6 months. The occurrence of AF comorbidities in patients was recorded monthly, as indicated in Figure 3.

### 2.8 Statistical analysis

Statistical analysis was performed using SPSS 27.0. Measurement data were expressed as mean ± standard deviation (x ± s), and count data were expressed as number of cases and percentage. Paired-samples t-test was used for pre- and post-treatment comparisons of measures that conformed to the normal distribution, and the Wilcoxon signed-rank test was used for pre- and post-treatment comparisons of measures that conformed to skewed distribution. Multiple group comparisons (at different current intensity stimulation) were taken as Friedman test. Statistical significance was defined as *p* < 0.05.

## 3 Results

### 3.1 General clinical characteristics of patients

A total of 22 subjects were included in this study, of which 14 (63.6%) were males. Among the previous disease history, 12 had chronic heart failure (54.5%). The rest of the information is shown in Table 1.

**Table 1:** General clinical characteristics of patients.

Characteristics	*n* = 22
Age(yrs)	73.95 ± 12.69
Male, No. (%)	14(63.6)
BMI (kg/m^2^)	24.70 ± 4.77
cigarette smoking, No. (%)	3(13.6)
CHA2DS2-VASc Score	3.23 ± 1.60
History of previous illnesses	
Chronic heart failure, No. (%)	12(54.5)
Coronary heart disease, No. (%)	10(45.5)
Hypertension, No. (%)	14(63.6)
Diabetes, No. (%)	6(27.3)
Hyperthyroidism, No. (%)	2(9.1)
Pulmonary Hypertension, No. (%)	12(54.5)
Medications taken	
Beta blockers, No. (%)	11(50.0)
Calcium channel blockers, No. (%)	12(54.5)
Statins, No. (%)	13(59.1)
Diuretics, No. (%)	9(40.9)

BMI, Body Mass Index; CHA2DS2-VASc Score, which suggests a stroke prevention risk score for patients with atrial fibrillation.

### 3.2 Comparison of blood pressure and heart rate before and after a single LL-aVNS treatment

The levels of SBP, DBP, HRmax, and HRmin decreased after a single LL-aVNS treatment and the difference was statistically significant (*p* < 0.05), as indicated in Table 2.

**Table 2:** Comparison of blood pressure and heart rate before and after a single LL-aVNS treatment.

Characteristics	Before	After	*p*
SBP(mmHg)	130.47 ± 15.05	128.03 ± 15.45	0.019
DBP(mmHg)	77.50 ± 12.13	75.16 ± 12.19	0.030
HRmax(*bpm*)	103.17 ± 20.62	100.04 ± 20.30	0.002
HRmin(*bpm*)	73.84 ± 12.43	69.91 ± 13.82	<0.001

SBP, systolic blood pressure; mmHg, the unit of measurement for blood pressure; DBP, diastolic blood pressure; HRmax, maximum heart rate: HRmin, minimum heart rate; bpm, the unit of measurement for heart rate.

### 3.3 Comparison of blood pressure and heart rate during stimulation with different current intensities in LL-aVNS

The stimulus current threshold of the 22 subjects in this study was 20.76 ± 2.83 mA, ranging from a peak of 25 mA to a low of 16 mA. Notable variances were observed in comparing SBP, DBP, HRmax, and HRmin under varying current intensities in the LL-aVNS (*p* < 0.05). The values of SBP, DBP, HRmax, and HRmin were the smallest when the stimulus intensity was 8 mA, as presented in Table 3.

**Table 3:** Comparison of blood pressure and heart rate during stimulation with different current intensities in LL-aVNS.

Characteristics	SBP (mmHg)	DBP (mmHg)	HRmax (*bpm*)	HRmin (*bpm*)
5 mA	125.34 ± 15.84 ab	72.31 ± 13.15 a	97.40 ± 19.25 a	68.89 ± 13.22 a
8 mA	124.84 ± 14.44 ab	72.31 ± 12.53 ab	97.11 ± 19.56 a	68.46 ± 13.58 a
10 mA	124.97 ± 13.76 ab	73.07 ± 12.40 a	97.40 ± 19.61 a	69.76 ± 13.09 a
15 mA	128.36 ± 16.05	75.31 ± 11.25	97.17 ± 19.56 a	69.41 ± 13.55 a
thresholds	129.93 ± 15.18	74.96 ± 13.36	99.44 ± 20.48	71.04 ± 13.26
*p*	<0.001	<0.001	0.008	<0.001

SBP, systolic blood pressure; DBP, diastolic blood pressure; mmHg, the unit of measurement for blood pressure; HRmax, maximum heart rate: HRmin, minimum heart rate; bpm, the unit of measurement for heart rate; a, *p* < 0.05 compared with the threshold group; b, *p* < 0.05 compared with the 15 mA group.

### 3.4 Comparison of blood pressure, heart rate, LVEF, and LAD before and after 4 weeks of treatment

The levels of SBP, DBP, and HRmin decreased after 4 weeks of treatment, and the difference was statistically significant (*p* < 0.05). There was no significant difference in the comparison of HRmax, LEVF, and LAD before and after treatment, as shown in Table 4.

**Table 4:** Comparison of blood pressure, heart rate, LVEF, and LAD before and after 4 weeks of treatment.

Characteristics	Before	After	*p*
SBP (mmHg)	134.75 ± 18.25	123.83 ± 14.88	0.014
DBP (mmHg)	81.08 ± 10.96	75.75 ± 12.79	0.044
HRmax (*bpm*)	106.66 ± 17.20	102.08 ± 17.84	0.246
HRmin (*bpm*)	74.80 ± 12.38	68.88 ± 10.30	0.008
LVEF (%)	55.05 ± 9.57	56.12 ± 7.69	0.085
LAD (mm)	45.16 ± 4.24	44.83 ± 4.01	0.314

SBP, systolic blood pressure; DBP, diastolic blood pressure; mmHg, the unit of measurement for blood pressure; HRmax, maximum heart rate: HRmin, minimum heart rate; bpm, the unit of measurement for heart rate; LVEF, left ventricular ejection fraction; LAD, left atrial diameter.

### 3.5 Comparison of AFSS and MSAS-HF symptom scores before and after 4 weeks of treatment

Symptom scores of AFSS and MSAS-HF after 4 weeks of treatment were decreased compared to before (*p* < 0.05), suggesting that patients’ symptoms were significantly improved compared to before treatment, as seen in Table 5.

**Table 5:** Comparison of AFSS and MSAS-HF symptom scores before and after 4 weeks of treatment.

Characteristics	Before	After	*p*
AFSS Palpitations Score	3.50 ± 1.34	2.18 ± 0.66	<0.001
AFSS Shortness of Breath At Rest Score	1.95 ± 1.43	1.64 ± 1.00	0.016
AFSS Post-Activity Shortness of Breath Score	2.86 ± 1.78	2.55 ± 1.50	0.016
AFSS Exercise Intolerance Score	2.82 ± 1.76	2.32 ± 1.32	0.002
AFSS Dizziness Score	2.27 ± 1.61	1.50 ± 0.80	0.001
AFSS Fatigue Score	3.45 ± 1.53	2.09 ± 0.75	<0.001
AFSS Chest Pain Score	3.09 ± 1.74	2.09 ± 0.87	<0.001
MSAS-HF Score	35.45 ± 9.31	20.82 ± 4.76	<0.001

AFSS, Atrial Fibrillation Severity Scale; MSAS-HF, Memorial Symptom Assessment Scale for Heart Failure.

### 3.6 Adverse effects

There was one patient who developed localized skin itching and skin erythema after scratching on the 2nd day after the first LL-aVNS treatment, and the itching was alleviated and the erythema subsided after treatment with a glycerite lotion. Other patients did not experience adverse reactions such as skin itching, headache, muscle twitching, or dyspnea. The study was followed up for a total of 6 months. Two patients were hospitalized for acute exacerbation of chronic heart failure between 4 and 6 months after treatment.

## 4 Discussion

The main findings of this study indicate that (a) LL-aVNS can effectively lower SBP, DBP, and HRmin in patients with paroxysmal atrial fibrillation; (b) LL-aVNS can help to alleviate symptoms and improve the quality of life; and (c) the process of LL-aVNS is safe and feasible.

### 4.1 Modulation of ANS activity

The cardiac autonomic nervous system (ANS) can be divided into extrinsic ANS and intrinsic ANS according to its anatomical location (Choi et al., 2010). Nerve fibers in the brainstem and before the ganglion plexus (GP) constitute the cardiac extrinsic ANS, while the GP located on the cardiac surface, near the great vessels, and the network of nerve fibers connecting the GP constitute the cardiac intrinsic ANS, and the role of the intrinsic cardiac ganglia(ICG) should not be ignored (Chadda et al., 2018; Squair et al., 2021). The cardiac intrinsic and extrinsic ANS are closely linked functionally and structurally and participate in the neurohumoral system to regulate cardiac functional activities (Herring et al., 2019). This regulation is mainly dependent on neurotransmitters released from sympathetic or vagal nerves in the GP. Through the binding of neurotransmitters to the corresponding receptors, functional changes in the ion channels of the atrial myocyte membrane are mediated (Armour, 2011).ANS remodeling is mainly manifested by enhanced ANS discharge activity or increased nerve density, which leads to an imbalance between the sympathetic and vagal nerves and triggers atrial fibrillation (Ardell and Armour, 2016). Furthermore, ICG contains noradrenergic and neurotrophic factors, and therefore, stimulation of the cardiac ANS may produce complex effects *in vivo* (Hoard et al., 2008). It is further hypothesized that modulation of ANS activity may be an important therapeutic strategy for the treatment of AF (Shen et al., 2011).

### 4.2 The mechanism of LL-aVNS

In previous studies, cardiac vagus nerve stimulation was always thought to result in a shortening of the atrial-effective refractory period (AERP) and an increase in the inducibility of atrial fibrillation (Liu and Nattel, 1997). However, in recent years, several studies have shown that low-intensity vagal nerve stimulation has a high value in the treatment of AF (Zhao et al., 2018; Salavatian et al., 2016; Ardell et al., 2014). Low-intensity vagus nerve stimulation can mildly slow down sinus tachycardia or atrioventricular conduction, significantly prolong the effective occlusion period, inhibit the triggering of atrial fibrillation, and shorten the duration of atrial fibrillation in the pulmonary veins and atrial sites (Li et al., 2009). It has been found that the auricular region is the only region with vagal afferent fiber distribution on the surface of the human body, and stimulation of the peripheral pathway of the auricular branch of the vagus nerve can regulate the activity of the brainstem, thalamus, cerebral cortex, and other related regions, inhibit sympathetic nerve activity, and thus restore the cardiac ANS homeostasis (Kraus et al., 2007; Shiozawa et al., 2014). Therefore, LL-aVNS has entered the researchers’ field of vision as a noninvasive vagus nerve stimulation modality.

In addition, the mechanism of LL-aVNS in the treatment of AF may be related to its specific inhibition of the inflammatory response. Inflammation is thought to be associated with the development and maintenance of AF, and elevated serum levels of inflammatory biomarkers, such as C-reactive protein, have been associated with the recurrence of AF after successful reentry (Yo et al., 2014). In one study, LL-aVNS was administered to 26 patients after cardiac surgery, which inhibited the inflammatory response induced by cardiac surgery and reduced the levels of inflammatory markers such as TNF-*α*, interleukin-6, interleukin-10, and C-reactive protein, with a consequent decrease in the incidence of atrial fibrillation, when compared with the control group (Stavrakis et al., 2017). Stavrakis et al. on the clinical control of LL-aVNS for the treatment of paroxysmal AF The study showed that both short-term (1 h) LL-aVNS and long-term (1 h per day for 6 months) LL-aVNS reduced the levels of inflammatory factors such as TNF-α (Stavrakis et al., 2015; Stavrakis et al., 2020).

### 4.3 BP and HR

Unlike the observables in the study by Stavrakis et al., the present study focused on the blood pressure and heart rate modulating effects of LL-aVNS and the assessment of clinical symptoms in paroxysmal AF treated with LL-aVNS in combination with drugs. Data from this study showed a decrease in SBP, DBP, HRmax, and HRmin after a single treatment with LL-aVNS and a decrease in SBP, DBP, and HRmin after 4 weeks of treatment. Tobaldini et al. showed that transcutaneous vagus nerve stimulation (tVNS) via the auricular branch of the vagus nerve decreased heart rate and affected cardiac and peripheral autonomic function (Tobaldini et al., 2019). A study by Antonino et al. also showed a significant decrease in resting heart rate during tVNS (Antonino et al., 2017). Furthermore, Zhu Haixia et al. conducted tVNS in patients with refractory hypertension and showed that tVNS had a modulating effect on diastolic and systolic blood pressure and heart rate (Haixia et al., 2022). These findings are consistent with the current study and suggest that vagus nerve stimulation may function to modulate blood pressure and heart rate in humans.

During the LL-aVNS of this study, the stimulus current threshold was 20.76 ± 2.83 mA in 22 subjects, ranging from a peak of 25 mA to a low of 16 mA. In the mode of operation with a pulse frequency of 1–120 Hz (randomly varied) and a pulse width of 0.2 ms, SBP, DBP, HRmax, and HRmin reached their minimum values when the stimulus intensity was 8 mA. Currently, LL-aVNS lacks a standard stimulation protocol (Zafeiropoulos et al., 2023), i.e., the stimulation parameters of LL-aVNS (e.g., stimulation frequency, pulse width, current intensity, etc.) are not consistently used in various studies. The present study provides a reference stimulation protocol and verifies that 8 mA may be the optimal stimulation intensity under this stimulation protocol. This may be related to its vagal excitation. Acetylcholine released after vagal excitation binds to M2 receptors on the membrane of atrial myocytes, antagonizing the effects of sympathetic excitation, producing a slowing of pacing frequency, slowing the autoregulation of Purkinje fibers, and slowing atrial conduction (Armour, 2011; Pickard et al., 2017).

In this study, the overwhelming majority of the enrolled patients were male. Nevertheless, it has been reported that women possess stronger cardiac-specific sympathetic activation (Mitoff et al., 2011). Consequently, we hypothesized that female patients may benefit more from LL-aVNS. We will focus on this meaningful point in future more in-depth studies.

### 4.4 AFSS and the MSAS-HF scale

Both the Symptom Scale of AFSS and the MSAS-HF Scale have been shown to have the ability to recognize clinically meaningful differences in symptoms (Streur et al., 2018; Zhou et al., 2022). The results of this study showed a significant decrease in the symptom scale score of the AFSS after 4 weeks of treatment compared to the previous one, suggesting that the patients’ AF-related symptoms were better than before and that their quality of life had improved. Investigations have shown that 33% of patients with paroxysmal atrial fibrillation suffer from congestive heart failure (Chiang et al., 2012). 54.5% of the patients included in the present study had chronic heart failure, therefore, the present study was also evaluated using the MSAS-HF scale, which showed a decrease in the scale scores from the previous period, and a significant improvement in the symptoms of heart failure. The studies of Stavrakis et al. and Kulkarni et al. both showed that after 6 months of LL-aVNS at 1 h per day, AF load was significantly decreased in the treatment group compared to the control group (Stavrakis et al., 2020; Kulkarni et al., 2021). This is similar to the significance of the results of the present study which showed that LL-aVNS can improve the clinical symptoms, suggesting that LL-aVNS can be applied to the treatment of paroxysmal AF with some clinical efficacy.

### 4.5 Adverse effects and personalized treatment

Except for one patient who developed localized itching of the skin after the first LL-aVNS treatment, none of the patients experienced adverse effects. During follow-up, two patients were hospitalized for acute exacerbation of chronic heart failure 4 to 6 months after treatment. This may indicate that the effects of short-term LL-aVNS are limited and that long-term LL-aVNS may be required. Previous studies have shown that the duration of activating or inhibitory effects elicited by nerve stimulation can greatly exceed the duration of the stimulation (Gomes-Osman et al., 2018) and that intermittent vagal stimulation also avoids neuromuscular fatigue (Lu et al., 2015). the minimum duration of LL-aVNS stimulation required to produce long-term effects has not yet been clearly defined. Based on the results of the present study, after 1 month of continuous LL-aVNS treatment, the time for another LL-aVNS treatment may need to be within 4 months. Of course, this may be related to the patients’ respective physical fitness and treatment compliance, further suggesting that LL-aVNS requires a personalized and targeted treatment regimen.

## 5 Conclusion

In this study, our findings revealed that for patients with paroxysmal atrial fibrillation, LL-aVNS not only aids in the regulation of blood pressure and heart rate but also effectively alleviates symptoms. Moreover, the treatment process was demonstrated to be safe and feasible. This study provided a validated LL-aVNS stimulation protocol with a randomly varying pulse frequency of 1–120 Hz, a pulse width of 0.2 ms, and an optimal stimulation intensity of 8 mA.

LL-aVNS can be a noninvasive, safe, effective, and economical adjunctive treatment option for paroxysmal AF. However, the sample size of this study was small and should be increased in the future to further validate the effectiveness. In addition, further studies are needed to optimize the individualized treatment for better application in clinical treatment. We believe that a smarter, portable, affordable, multi-functional test with accurate results, medical or home wearable low-intensity auricular vagus nerve stimulator will better promote this treatment technology.

## Data Availability

The original contributions presented in the study are included in the article/supplementary material, further inquiries can be directed to the corresponding authors.
